# Electrothermal discharge by exploding of copper wires with different diameters

**DOI:** 10.1038/s41598-025-11130-7

**Published:** 2025-09-12

**Authors:** F. B. Diab, M. A. Abd Al-Halim, M. E. Abdel-kader

**Affiliations:** 1 Plasma and Nuclear Fusion Department, Nuclear Research Center, Egyptian Atomic Energy Authority, Nasr City, 13759 Egypt; 2 Physics Department, Faculty of Science, Benha University, Benha, 13518 Egypt

**Keywords:** Wire explosion, Wire diameter, Resistivity, Specific heat, Latent heat, Thermal expansion, Time of explosion, Materials science, Physics, Energy science and technology

## Abstract

This paper explores the explosion behavior of copper wires with diameters between 0.16 mm and 0.50 mm, using an electrothermal discharge system comprising capacitors of total energy up to 1.11 kJ. To improve accuracy, this study incorporates the effects of thermal expansion, resistivity, specific heat capacity, and phase transformation. The variations of the different electrical properties of the copper wires were calculated as a function of the discharge time, wire temperature, and wire diameter. The temperature profile for copper was based on the melting point (1357 K) and boiling point (2835 K). The Shomate equation was used to calculate the specific heat capacity of the copper wires as a function of temperature; while the wire resistivity was determined using a fitting equation based on previous published data. The results indicate that the time needed for wire explosion decreases for the higher charging voltage, while it increases with larger wire diameter. The thermal expansion shows a 9.95% increase in length and a 19.9% increase in cross sectional area. Additionally, as the discharge occurs, the wire temperature rises, causing an increase in both of the specific heat and the wire resistivity. Furthermore, the specific action integral converges to a constant value of approximately 2.1 × 10^9^ A^2^ s/cm^4^ for all diameters.

## Introduction

Electrothermal discharges generated by pulsed power facilities have a significant importance in basic research and applications. The wire explosion experiment is of particular interest because it is linked to several applications which are heavily related to the diverse physical properties of the involved materials. This method is considered as a platform to test the materials according to their different properties which may vary under extreme conditions dependent on wire properties.

The electrical wire explosion technique was used effectively for the synthesizing of nanomaterials. For example, combined silver/copper nanoparticles where synthesized with diameters found to be affected by the applied voltage^1^, as well as the thermal conductivity of carbon dioxide and methane media during the formation of the nanoscale tungsten carbide particles^2^. The complete vaporization of the silver wires was found to be essential to ensure the production of nano powder and avoid the formation of micrometer size particles^3^. Also, it was found that the gas pressure influences the size of the aluminum nanoparticles produced via the explosion of aluminum wire^4^.

Nano size tungsten oxide was synthesized via wire explosion process in low oxygen pressure, and the process was detected using high speed camera^5^. Besides, other nanoparticle materials such as the molybdenum carbide^6^ and the zirconium nitride^7^ could be produced.

Simple analytical models were developed to characterize the wire exploding process based on the temperature-dependent properties of wire, such as the latent heat, the resistivity, and the specific heat^5–7^. A thermodynamic calculation approach was applied to describe the heating mechanism during the wire explosion of several wire material (Al, Cu, Ni, Fe, Mo, and Ti), involving the resistivity and the specific heat as a function of the wire temperature, and it showed good agreement when compared with experimental results^8^. It is worth indicating that the peak voltage observed in the slow-rate explosion was higher than that in the fast-rate explosions^9^. In most experiments, the break point is characterized by a spike in the discharge voltage signal or by a sudden drop in the discharge current signal^2–6^. Additionally, the explosion process could also be visualized using high-speed photography^7^.

Copper, aluminum, and nickel wires are commonly used in the explosion experiments, either in air and underwater. For instance, Cu wire of 5 cm in length and 0.3 mm diameter requires 194 J to be vaporized during the wire explosion underwater^10^. In air medium experiments, Cu and Ni wires with diameters of 25 and 50 μm were electrically exploded and the increase of the wire diameter due to thermal expansion was observed^11^. The Cu/Ni wire alloy requires a longer initial deposited energy rate due to its higher resistivity^12^. This can be generalized to any metal to show how greater electrical resistance can lead to increased energy deposited.

The explosion process was analyzed using the target time-of-flight method combined with optical measurement^13^, while fast photography and x-rays radiography were used to investigate the fragmentation phases and the plasma development^14^. Furthermore, a piezoelectric gauge was used to detect the generated shock waves when Cu wires of 200 μm diameter and 5 cm long were exploded using different input energies in range between 70 J and 160 J^15^. Additionally, various wire materials (Cu, Al, Mo, and W) were implanted to explode and generate shock waves^16^. There are two ways in which the explosion process can be explained^17^. For refractory material wires such as tungsten, the breakdown occurs at the boundary layer between the surrounding medium and the dense products of the explosion material, where the conductive layer moves in direction tracking the shock wave front. For wires with a low melting point such as copper, the breakdown begins within the region of the explosion products, rather than the surrounding medium^17^.

Besides, the optimization of the wire radius and charging voltage can enhance the specific energy deposition, and the action integral is influenced by current rise rate and the wire diameter ^18^. A low-capacitance system operating in high charging voltage cause the discharge to occur over a short period of time, resulting in a higher current rise rate, which causes a rapid and intense heating. Thus mechanism is much efficient for the wire vaporization when using the same stored energy, yielding more stable explosion and enhanced the shock wave generation^19^. The stable explosion can be achieved using a high charging voltage at a low capacity, even using the same stored energy with different capacities, which results in more efficient energy deposition during the wire evaporation phase, due to the greater magnetic stress associated with a shorter discharge time^19^.

While previous studies have not addressed the effect of wire diameter on the homogeneity of the generated nanoparticles, our study introduces the factors influencing this. Similarly, some other researches have often assumed constant wire dimensions, neglecting the role of thermal expansion. Although the change in resistance and specific heat with wire temperature has been studied previously, they have been studied separately and have not analyzed in conjunction study with changes in wire diameters used.

The present work combines multiple factors that might previously studied separately, practically or theoretically, or were completely neglected on other times. We present a semi-analytical analysis that incorporates the temperature-dependent variations of the thermal expansion, density, specific heat, and the electrical resistance, all with changes of the wire diameter. The analysis employs equations, whose coefficients and sources are officially documented, to achieve a formula offers better accuracy. This research can build a baseline for future experimental work to study the plasma formation under various conditions, practically and theoretically.

## Experimental setup

**Fig. 1:**
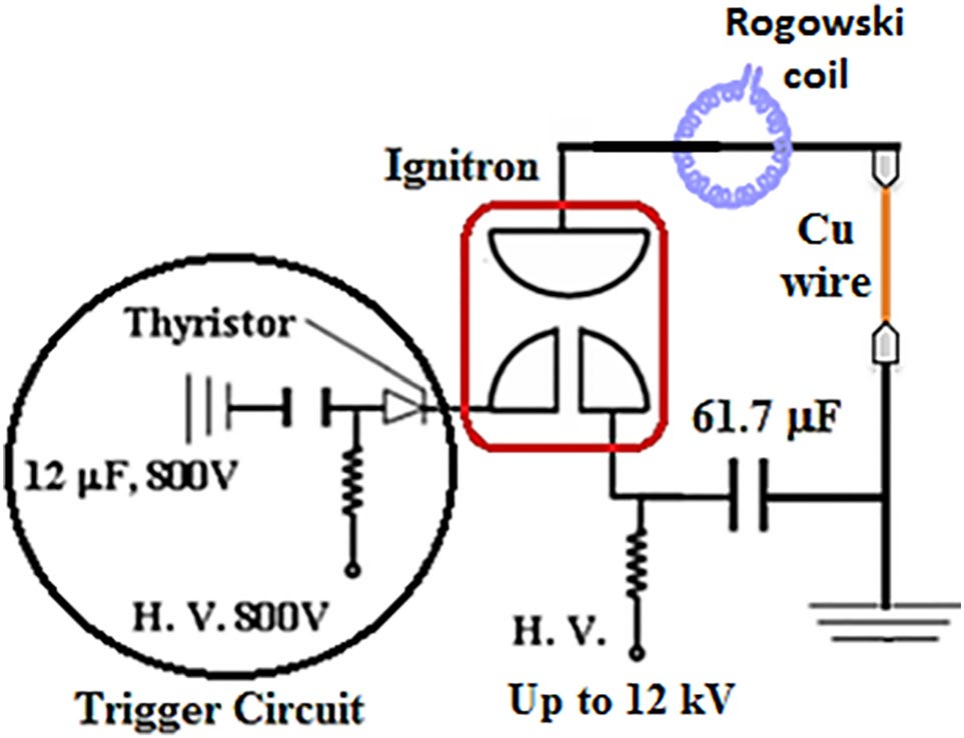
Schematic diagram of the discharge circuit.

The circuit featured a simple charging - discharging circuit as shown in Fig. 1, comprising a high voltage power supply which provides input voltage up to 12 kV, connected to a capacitor bank of 61.7 µF capacitance, via an ignitron switch for controlled discharge. The copper wires (2 cm length ) with five different diameters (0.16, 0.25, 0.30, 0.40, and 0.50 mm) were mounted between the terminal edges, to ensure good electrical contact and avoid any errors due to the bad connection. The charging voltage was adjusted between 2 and 6 kV to study the effect of different input energies. The discharge voltage was measured using a potential divider while the discharge current was measured using a calibrated Rogowski coil with an integrator circuit. All signals were recorded using digital oscilloscope of 100 MHz bandwidth which is adequate to needed value of about 2 MHz.

## Results and discussion

**Fig. 2:**
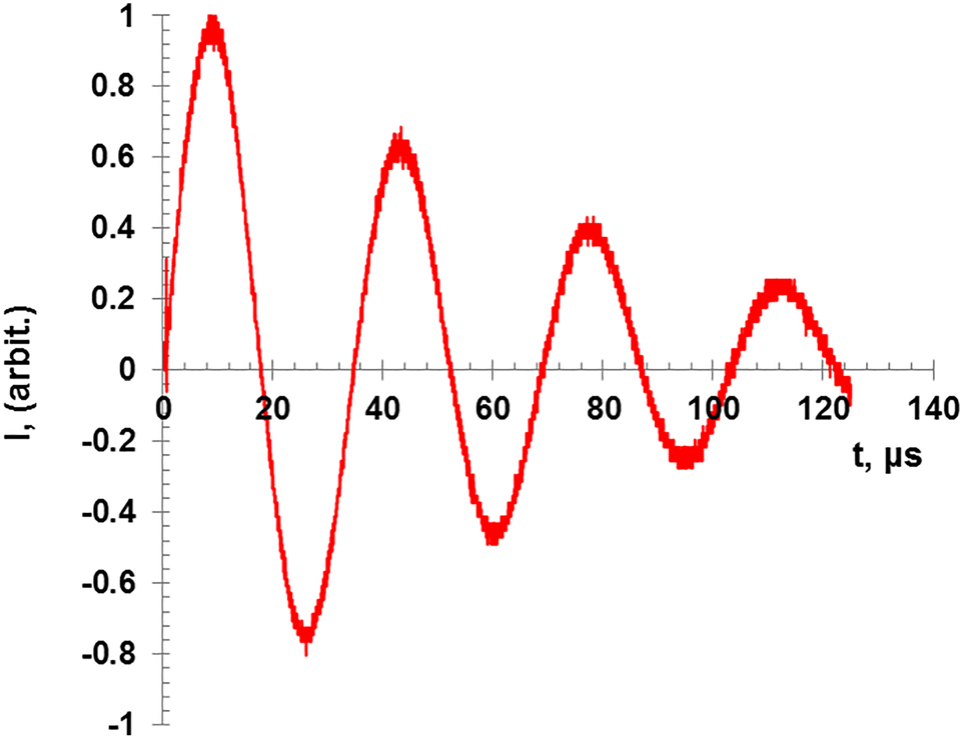
Discharge current signal of short circuit for 2 kV charging voltage.

The discharge current signal for the short circuit is shown in Fig. 2 and it is described by the RLC circuit equations. The discharge current is given as^20^:

1 $$I = I_{0} \sin \left( {\omega t} \right).\exp \left( {{-}R_{0} t/2L_{0} } \right)$$

where I_0_ is the first peak value of the discharge current, R_0_ is the total circuit resistance, and L_0_ is the total circuit inductance, which can be determined from the approximated resonance relation:

2 $${\upomega\:}{\text{L}}_{0}=\frac{1}{{\upomega\:}{\text{C}}_{0}}$$

where C_0_ is the capacitance of the capacitor bank. At the current peak i.e. when sin(ωt) = 1, it is denoted that I = I_p_ so the relation becomes:

3 $$\ln \left( {I_{p} /I_{0} } \right) = {-}R_{0} t/2L_{0}$$

In the present work, a Rogowski coil with an integrator is employed to measure the discharge current. The peak discharge current in the pulsed discharge is related to the charging voltage V_ch_ according to the relation^20–22^:

4 $$\:\text{I}=\frac{\uppi\left(1+\text{f}\right){\text{C}}_{0}{\text{V}}_{\text{c}\text{h}}}{{\text{T}}_{0}}$$

where T_0_ is the periodic time of the signal and f is the reversal ratio which is defined in terms of the sequential discharge current peaks, I_1_, I_2_, … etc. as^20–22^:

5 $$\:\text{f}=\frac{1}{4}\left(\frac{{\text{I}}_{2}}{{\text{I}}_{1}}+\frac{{\text{I}}_{3}}{{\text{I}}_{2}}+\frac{{\text{I}}_{4}}{{\text{I}}_{3}}+\frac{{\text{I}}_{5}}{{\text{I}}_{4}}\right)$$

6 $$\:{\text{R}}_{0}=-\left(\frac{2}{{\uppi\:}}\right)\left(\text{l}\text{n}\:\text{f}\right){\left(\frac{{\text{L}}_{0}}{{\text{C}}_{0}}\right)}^{0.5}$$

The experimental analysis shows that L_0_ = 475 nH and R_0_ = 12.4 mΩ.

**Fig. 3:**
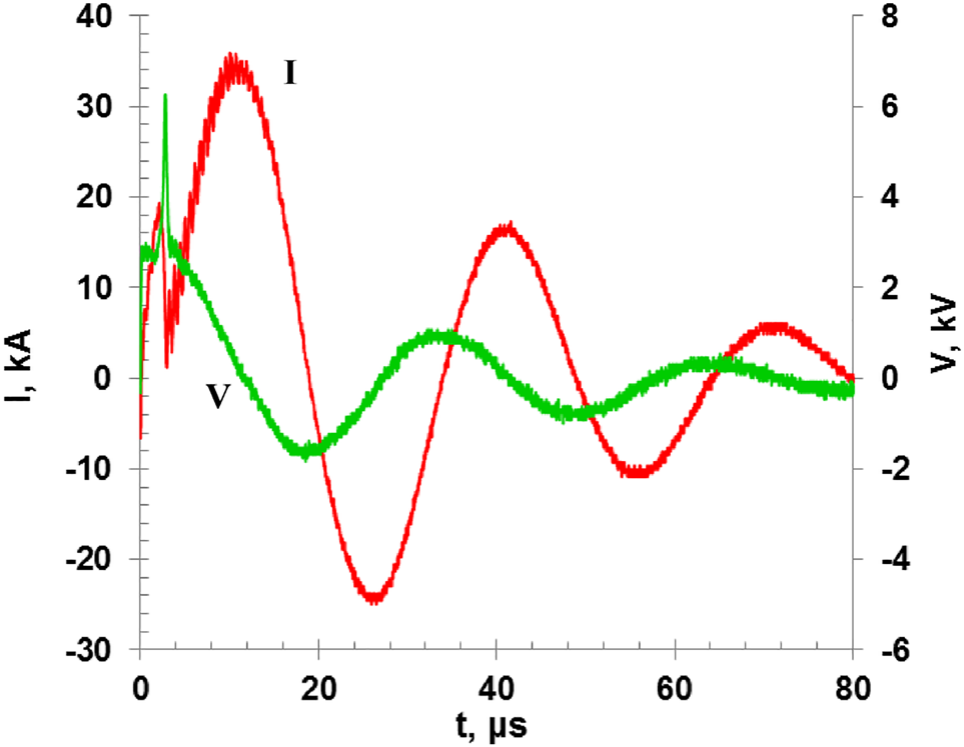
I-V Characteristic curves for 4 kV charging voltage and using 0.25 mm Cu wire diameter.

Figure 3 represents the discharge current and voltage waveforms which are recorded during the wire explosion. The discharge current signal exhibits a sinusoidal profile with a characteristic abrupt dip which refers to the point at which the load impedance reaches maximum during the explosion process takes place, which is corresponding to the vaporization of the wire, particles ionization, and formation of the plasma state. At the same time, the discharge voltage increases abruptly and forms a distinct spike^2–6^. Both the current dip and the voltage spike refer to the transition of the wire material into an ionized gas state that has a lower conductivity compared to the solid copper wire, which in turn reduces the current and raises the voltage needed to sustain the discharge.

Since the resistivity of the discharge region increases significantly due to the fragmentation/vaporization of the wire by the high-temperature, the current path will encounter a high resistance region. Then it is expected that the discharge voltage will rise correspondingly.

**Fig. 4:**
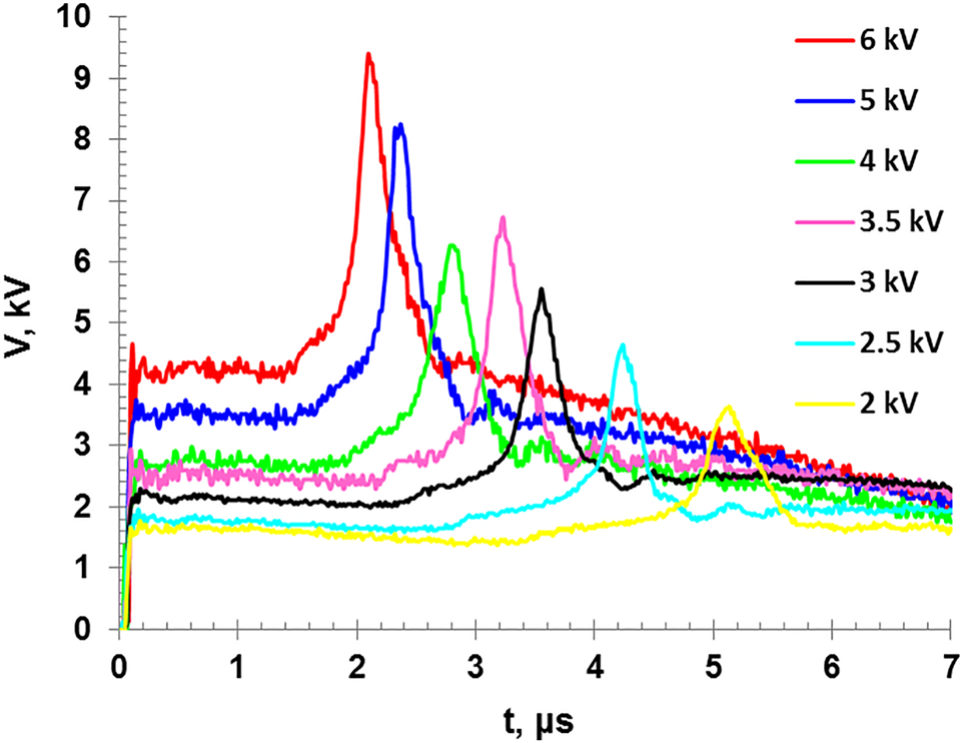
The discharge voltage as a function of the discharge time for different charging voltages.

**Fig. 5:**
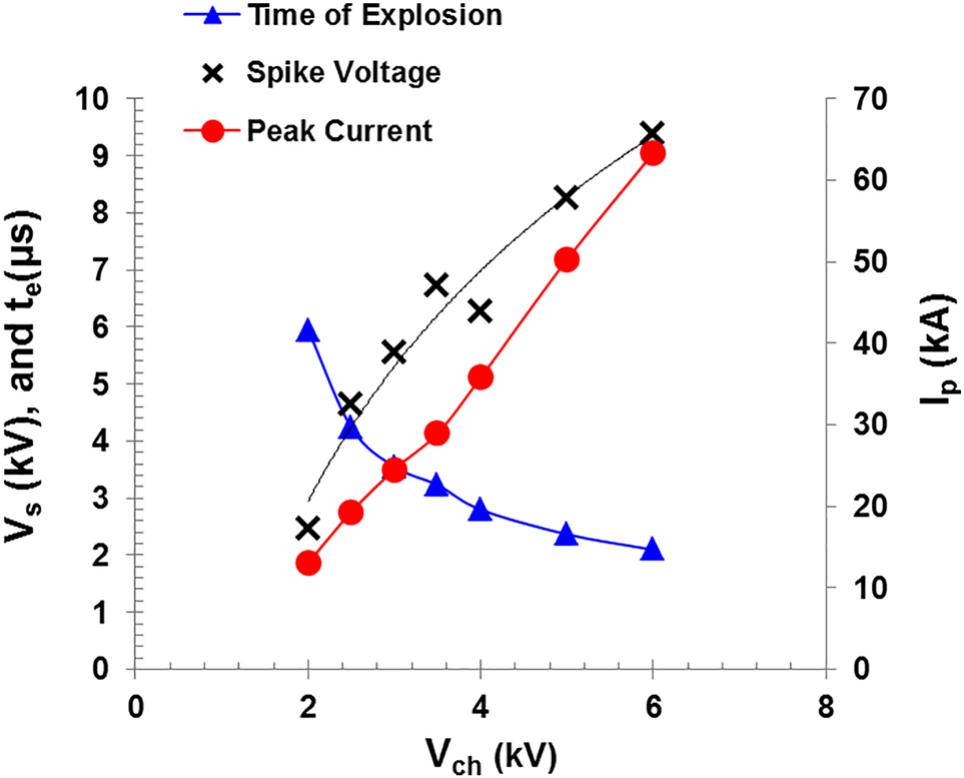
Variation of the peak current, the explosion time, and the spike voltage as a function of charging voltage for 0.25 mm Cu wire diameter.

An important aspect of such experiments involves comparing the discharge rates, by employing different capacitances to obtain slow or fast discharges^9^, or varying the input voltage while maintaining a constant capacitance as in the present study. We observe in Fig. 4 that the spike voltage increases, and the explosion time of the wires decreases as the charging voltage increased. Figure 5 shows that both the peak discharge current (*I*_p_) and the spike voltage (*V*_s_) increase with increasing the charging voltage while the explosion time (t_e_) is decreased. By this time, the evaporated wire atoms are ionized and the plasma state is formed.

**Fig. 6:**
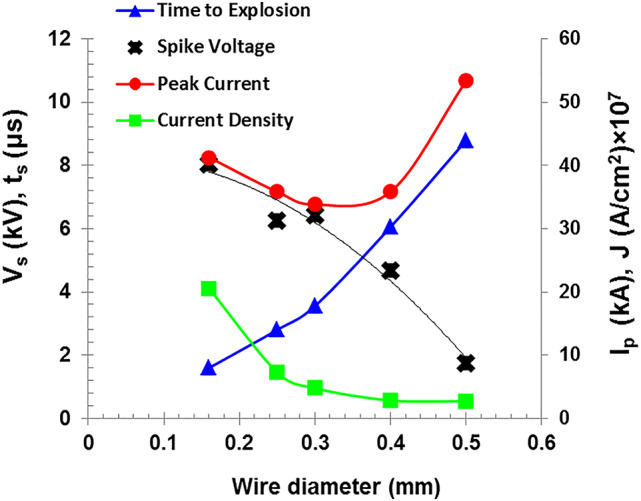
Time of explosion, spike voltage, Peak discharge current, and Peak current density as a function of Cu wire diameter for 4 kV charging voltage.

Increasing the wire diameter typically causes a higher peak discharge current. But, for thin wires, the exploding time is closely coinciding with the peak time of the discharge current, so it reduces the observed peak value of the discharge current, which explains the trend shown in Fig. 6, in which the peak of the discharge current decreases with increase in the wire diameter as the explosion time approaches the peak current time, before it is raised again. Furthermore, it is noticed that the explosion time of the Cu wire shows a clear dependence on diameter affected by two factors. First, the greater wire mass of the thick diameters requiring higher vaporization energy, and second, the current density which is lower in the thicker wires. Both factors causes that the thin wires reaches explosion faster due to its less mass and higher current density, while the thicker wires require longer time to reach the melting and evaporation temperature due to the greater mass and lower current density as illustrated in Fig. 6, which is consistent with other established researches.

One also observes that the voltage behavior varies for each wire diameter, where the smaller diameter wires exhibit a higher spike voltage even though the same used input voltage as shown in Fig. 6. This observation is due to the increased resistance of thinner wires, which elevates the voltage.

The energy deposited through the explosion during time dt is given as:

7 $$\text{E}=\int\:\text{I}\left(\text{t}\right){\text{V}}_{\text{w}}\text{(t)}\text{d}\text{t}$$

8 $$E = \int {I^{2}  (t) \rho \left( T \right)} \frac{\ell }{\delta }dt$$.

where, I(t) is the discharge current, V_w_(t) is the discharge voltage on the resistive wire, ρ(T) is the electrical resistivity as a function of temperature T, ℓ is the wire length, (the initial wire length ℓ_0_ (fixed to be 2 cm for all cases), and δ is the wire cross sectional area varied with time ($\updelta_{0}=\uppi\:r_0^2$ is the initial cross sectional area, and r_0_ is the initial wire radius). Also, the variation of the wire temperature ΔT is given as^8^:

9 $${\Delta\:}\text{T}=\frac{{\Delta\:}\text{E}}{\text{m}\text{S}\text{(T)}}$$

where m is the mass of the wire, S(T) is the specific heat as a function of the wire temperature.

The specific heat S(T) is divided according to the metal phase into two types S_S_(T) and S_L_(T), where S_S_(T) is the specific heat of the wire material in the solid state as a function of temperature, and S_L_(T) is the specific heat in the wire material in the liquid state as a function of temperature. The quantity of heat required to vaporize (explode) the wire could be given as^5,7^:

10 $$Q = mS_{S} \left( T \right)\Delta T + mL_{f} + mS_{L} \left( T \right)\Delta T + mL_{v}$$

where L_f_ is the latent heat of fusion (206 kJ/kg), L_v_ is the latent heat of vaporization (5071 kJ/kg).

**Fig. 7:**
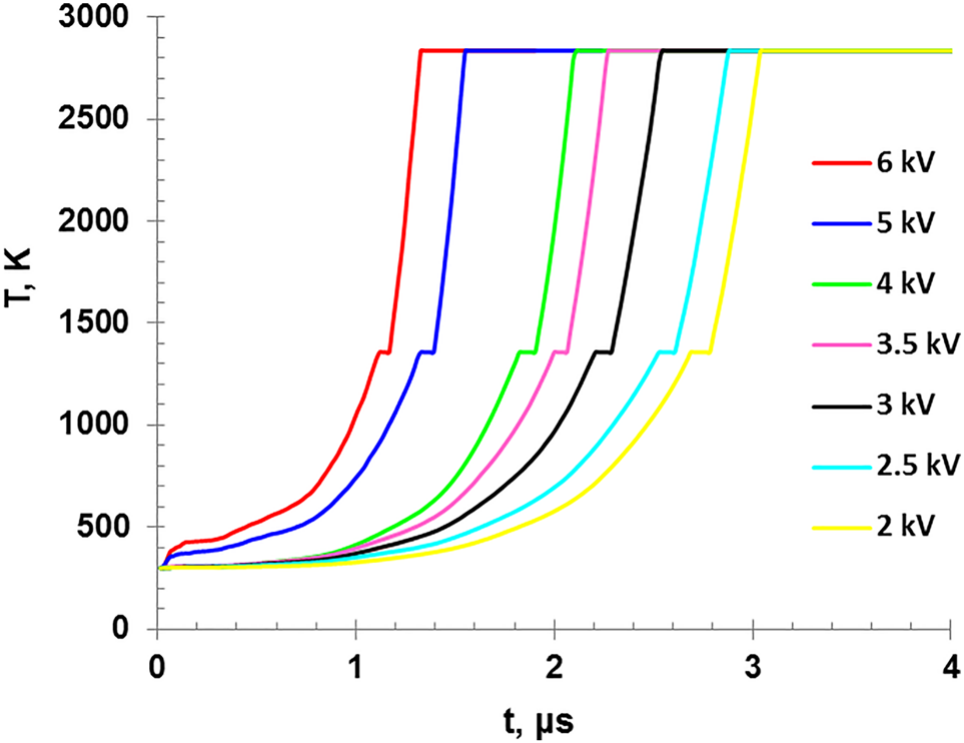
The variation of wire temperature as a function of time for different charging voltages.

**Fig. 8:**
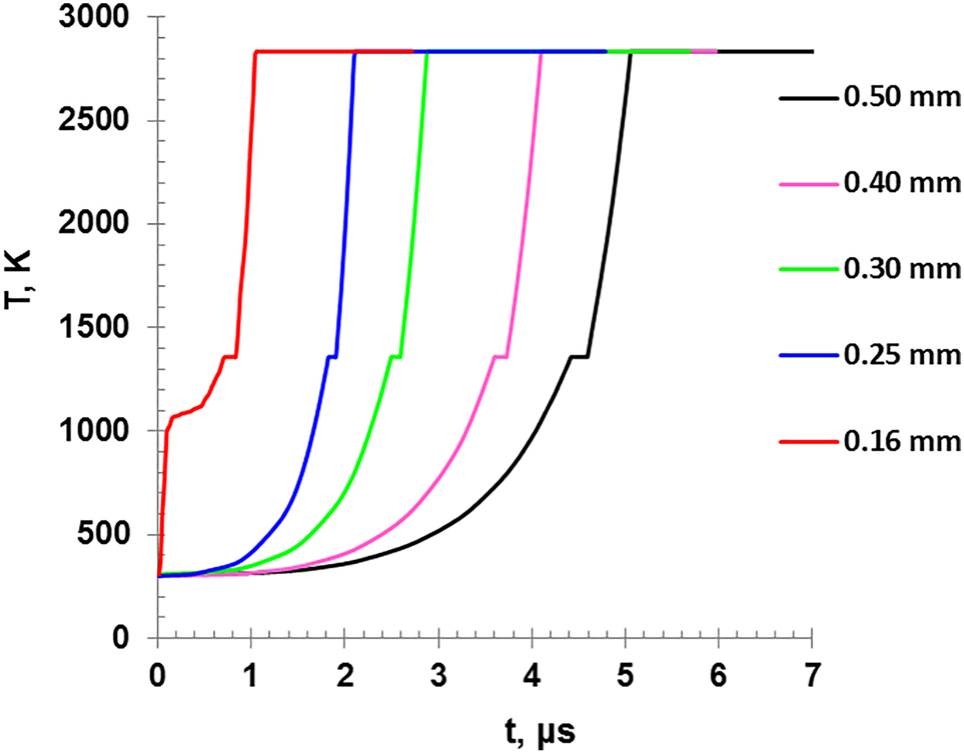
The variation of the wire temperature as a function of the discharge time for different wire diameters.

Figure 7 demonstrates the variation of the calculated wire temperature versus the discharge time which indicates that for lower charging voltage, a longer duration is needed to reach the melting (1357 K) or boiling points(2835 K). The higher input energies by ohmic heating reduce the time of explosion. Both Figs. 7 and 8 exhibit two distinct plateaus of the temperature. The first is corresponding to the melting point at which the temperatures remains unchanged as the wire is transforming from solid to liquid at T = 1357 K, while the second occurs during the boiling point when the wire material is transferred from liquid phase into vapor phase at T = 2835 K.

The S_S_(T) value is applicable only when the wire is still in the solid phase and its temperature is below the melting point of the copper (T_melt_ = 1357.6 K), that is T < T_melt_. In this temperature regime, the Shomate equation can be used to determine the specific heat capacity of the copper in solid phase (in unit of J/mol K) as^23^:

11 $$S_{S} \left( T \right) = A_{1} + A_{2} \tau + A_{3} \tau ^{2} + A_{4} \tau ^{3} + A_{5} /\tau ^{2} ~$$

where the fitting coefficients are given as A_1_ = 17.72891, A_2_ = 28.0987, A_3_=-31.25289, A_4_ = 13.97243, A_5_ = 0.068611, and τ is given as a function of the temperature in Kelvin, T(K) as: τ = T(K)/1000.

On the other hand, when the wire temperature reaches T_melt_, it remains unchanged for a short time until complete phase transformation of the wire material occurs. After that, during the liquid phase and below the boiling point (T_boil_) which is approximately 2835 K (i.e. T_melt_< T< T_boil_), the specific heat S_L_(T) should be used instead of S_S_(T), where S_L_(T) = 32.84 J/mol K (517.22 J/kg K)^23^.

**Fig. 9:**
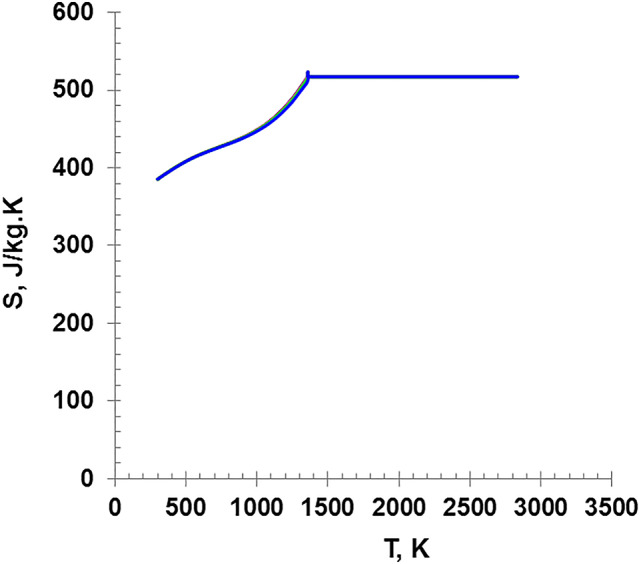
The variation of the specific heat as a function of the wire Temperature.

Figure 9 illustrates the variation of specific heat with temperature, according to the values of S_S_ and S_L_ as calculated in previous equations reference^23^. The specific heat of wire in its solid phase increases with temperature according to the Shomate equation. Then, after reaching the melting points, it maintains plateau until vaporization occurs. This trend is the same for all wires of the different diameters.

**Fig. 10:**
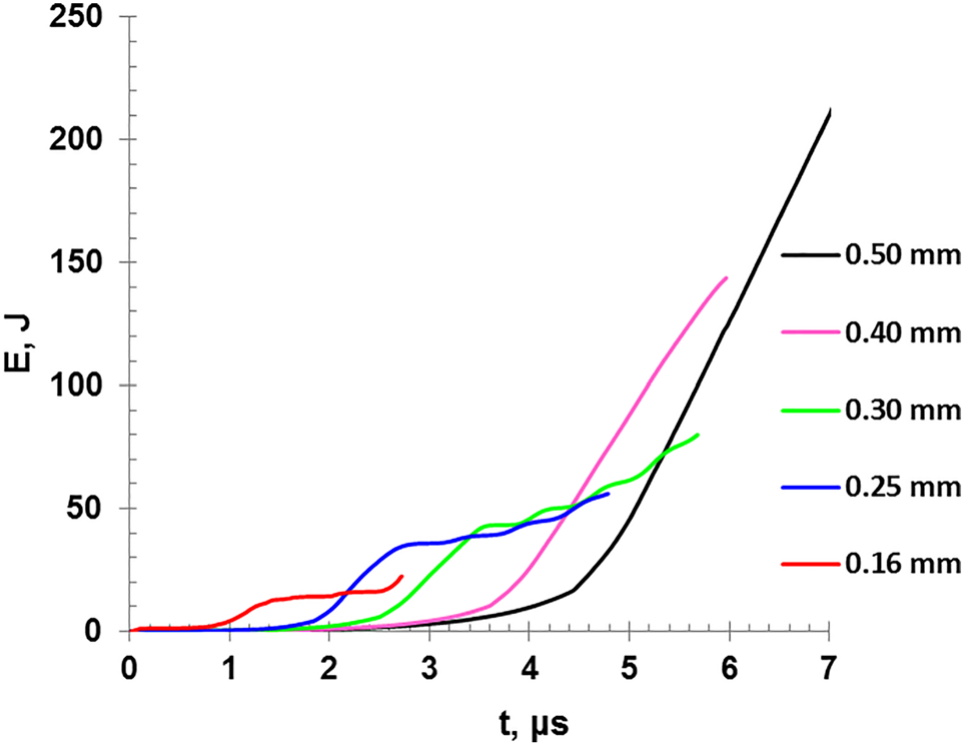
The variation of the deposited energy as a function of the discharge time for different wire diameters.

The variation of the deposited energy as a function of the discharge time for different wire diameters is shown in Fig. 10. It is shown that the thick wires need more energy and longer time to be transferred from solid phase into the liquid phase and then to explode and transfer into the vapor phase. The plateau-like in deposition energy (Fig. 10) matches other studies^15^, which validate the present analysis.

The analytical approach in the next sections used established thermodynamic analysis^8^, by incorporating the variation of the wire dimensions by thermal expansion, which affects the resistivity and other related parameters, to achieve the most precise calculations and the best possible accuracy. The thermal expansion ratio varies as phase changes. The variation of the wire length in the range between T = 293 K and the melting point can be described according to the fitting relation^24,25^:

12 $$(\Delta \ell /\ell _{0} ) \times 10^{2} = 1.685 \times 10^{{ - 3}} \left( {T - 293} \right) + 2.702 \times 10^{{ - 7}} \left( {T - 293} \right)^{2} + 1.149 \times 10^{{ - 10}} \left( {T - 293} \right)^{3}$$

where ℓ_0_ is the initial length of the wire, and ΔL the change in length.

On the other hand, for temperature between the melting point and the boiling point (T_melt_< T< T_boil_), the variation of wire dimensions can be calculated using the framework of Ref.^26^ which provides wider temperature coverage as compared to other researches^27^. In this regime, the thermal expansion could be determined via the relationship between density and temperature, where the wire density D, is given as a function of the temperature as^26^:

13 $$D = 9.077 - 8.006 \times 10^{{ - 4}} T$$

Also, the change of the copper wire density ΔD is given as:

14 $$\frac{{{\Delta \text D}}}{{{\Delta \text T}}} = - \gamma {\text{D}}$$

where γ is the coefficient of volume expansion of copper. Therefore, the coefficient of linear expansion of copper α could be calculated as α = γ/3, and it can be expressed as a function of the wire temperature using the fitting relation:

15 $$\alpha = 28.746 \times 10^{{ - 6}} \times \exp \left( {1.09 \times 10^{{ - 4}} T} \right)$$

**Fig. 11:**
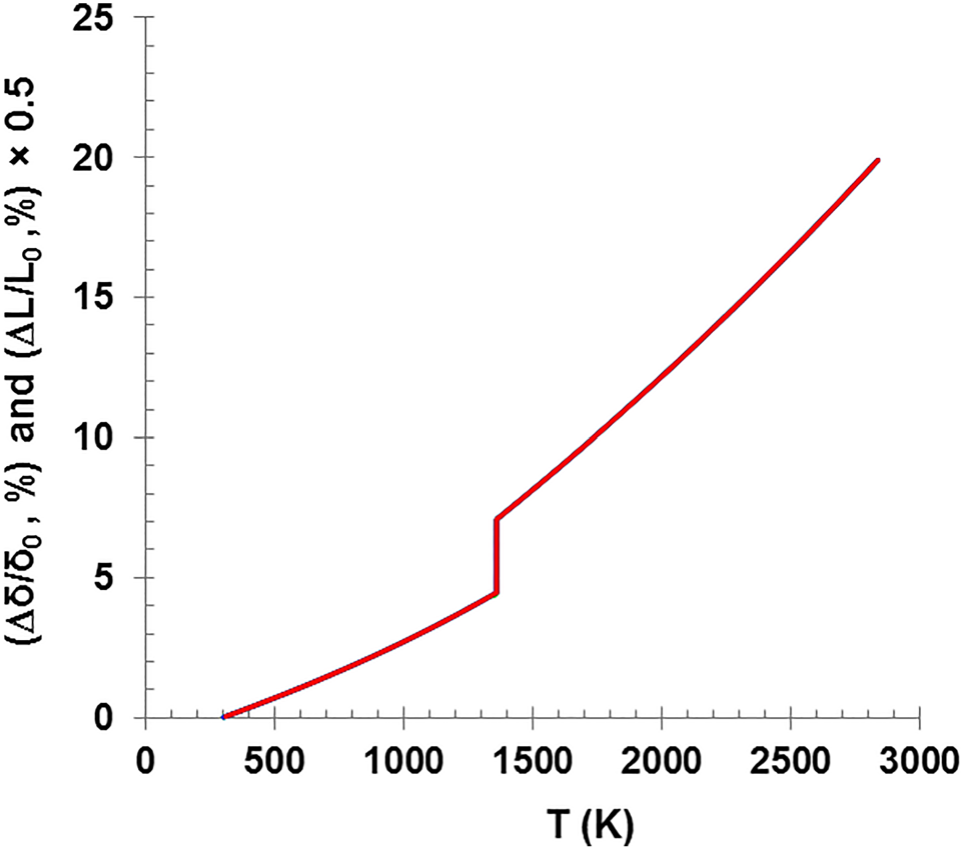
The change of the wire length and cross-sectional area as a function of the wire temperature.

**Fig. 12:**
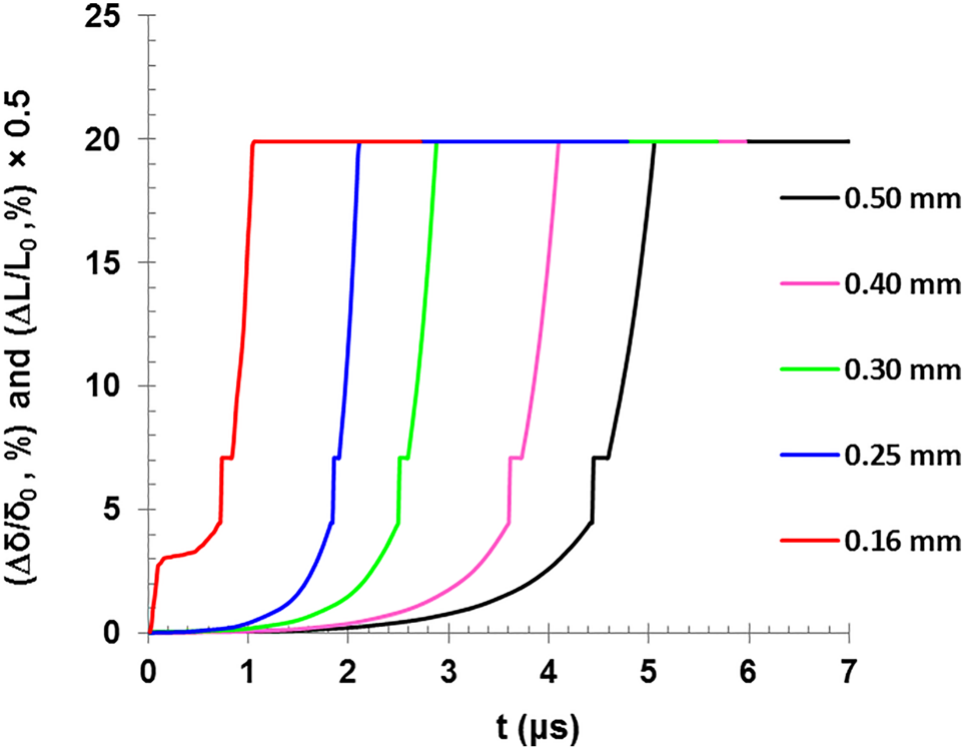
The change of the wire length and cross-sectional area as a function of the discharge time for different wire diameters.

Figure 11 shows the normalized dimensional changes for both the wire length (Δℓ/ℓ_0_) and cross-sectional area (Δδ/δ_0_) as a function of temperature. The cross-sectional area is expanded with about 19.9% while the length is expanded with about 9.95% which is half of the radial expansion. This result was obtained by applying both equations of thermal expansion (Eqs. 12 and 15) in the temperature range between 273 K and 2835 K. Figure 12 also corroborates the last findings, as the change in dimensions (area and length) is clearly shown with time as the temperature of the various diameter wires increases. It also shows the change of rate time required for the explosion to occur, in addition to the two plateau points at the melting and boiling temperatures.

The specific action integral h, can be used to analyze the process of wire explosion^28–31^. This parameter, can be given as:

16 $$h = \int {j^{2} \left( t \right)dt} = \int {\frac{{I^{2} \left( t \right)}}{{\delta ^{2} \left( t \right)}}dt} .$$

**Fig. 13:**
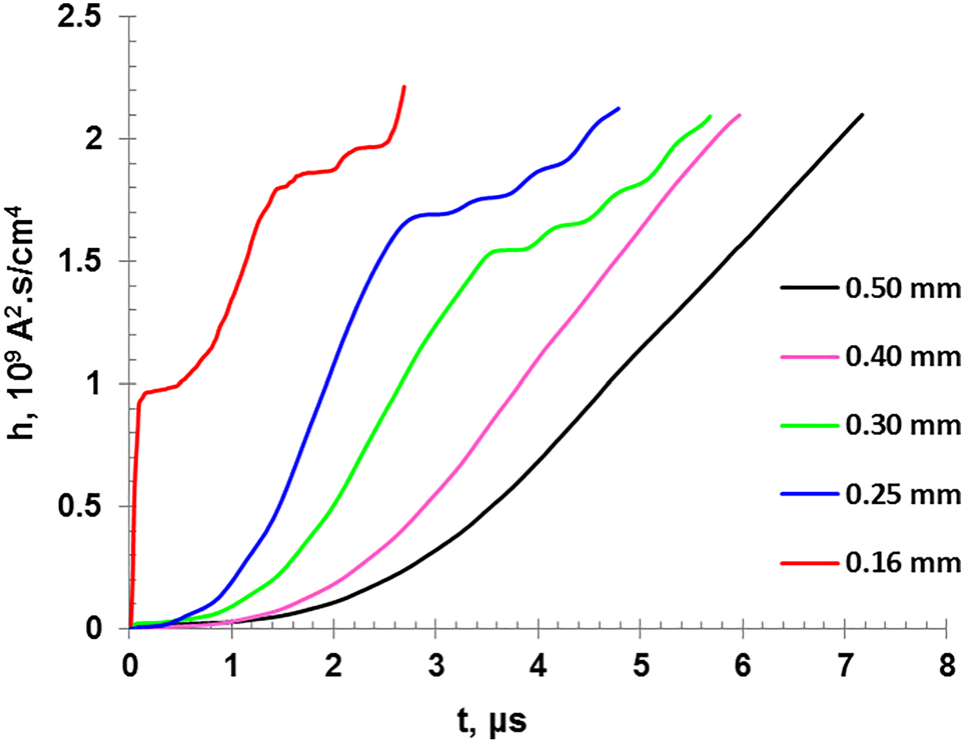
The variation of the specific action integral for different wire diameters as a function of the discharge time up to burst point.

Figure 13 represents the variation the specific action integral for different copper wire diameter, ranging from 0.16 mm to 0.50 mm, where all curves originate from zero since no current flows, then as time progresses, it increases. Thinner wires, exhibit a faster rate of increase in the specific action integral over the discharge time because of the higher current density which heats the lower wire mass in shorter time. While the rest of the wires show different explosion times as their diameter differ, they all converge to reach the same specific action integral value of about 2.1 × 10^9^ A^2^ s/cm^4^, and then they turns into a plasma state, regardless of the wire diameter.

**Fig. 14:**
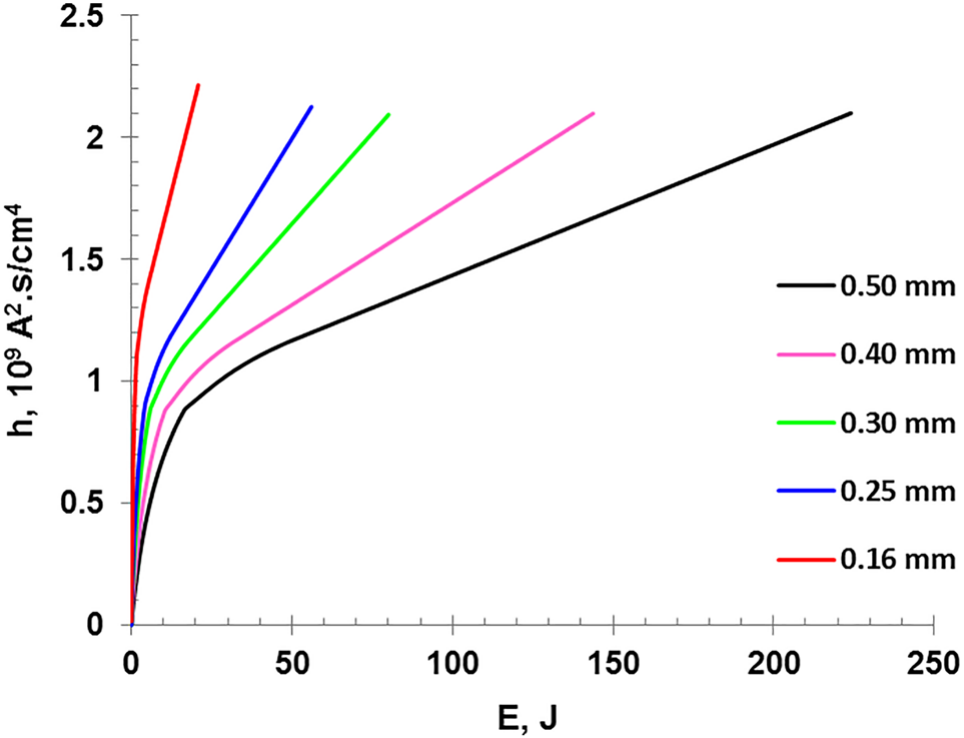
The specific action integral as a function of the deposited energy for different wire diameters.

Figure 14 demonstrates the variation of the specific action integral as a function of deposited energy. One observes that that thicker wires require more energy to explode, so the specific action integral is higher than that for thinner wires. Besides, all curves exhibit to have two different regimes, showing a steep slope at the beginning, which is corresponding to quick phase transition by rapid Ohmic heating. In the late regime, the rate of current rise stabilizes, when the current density reaching a lower rate of increasing near the current peak. It is concluded that a faster phase transitions is indicated in the early regime, while the late regime indicates accumulation of the deposited energy at the vaporization period.

The specific action integral is considered as a key of the thermal stability of metals under the influence of high currents in explosion regime^28,29^ and it is widely considered an intrinsic property of the material^28,29^. However, recent experimental studies showed that this assumption is valid only for high current densities up > 10^8^ A/cm^2^ or slightly higher^28,29,31^. Beyond these values, the specific action integral seems to be influenced by different factors, including the metal wire dimensions, pulse characteristics, circuit components and the discharge conditions^28,30,31^.

The present study used copper wires of fixed length and different diameters. Therefore, the calculated specific action integral is almost constant because it is still as a physical property of the material and it is related to the energy required convert the wires into vapor state. The action integral can also be expressed as:

17 $$h = \frac{{D^{2} \ell ^{2} }}{{m^{2} }}\int {I^{2} \left( t \right)dt} $$

It is concluded that the specific action integral remains intrinsic material-invariant regardless the wire mass and diameter. The action integral represents the amount of energy per unit mass which is required to completely convert the copper wire into vapor.

The cases which reporting that the specific action integral is varied with the discharge current, voltage, or wire diameter likely refer to experiments of non-ideal effects, such as incomplete vaporization. In the present study, which is a resistive heating one, the specific action integral converge to about 2.1 × 10^9^ A^2^ s m^−4^ kg^−1^, despite using thin or thick wires which reaches the same value of h but in different times.

Increasing the wire cross-sectional area reduces the wire resistance according to the law:

18 $$\:\text{R}\left(\text{T}\right)=\rho\left(\text{T}\right)\frac{\ell}{{\updelta\:}}$$

The electrical resistivity ρ(T) for T ≤ T_melt_ follows the relation in Ref.^32,33^, and then it is fitted as:

19 $$\rho \left( T \right) = 9.639 \times 10^{{ - 10}} T^{3} - 7.317 \times 10^{{ - 7}} T^{2} + 6.945 \times 10^{{ - 3}} T - 3.195 \times 10^{{ - 1}}$$

While for T_melt_< T≤T_boil_ the data in Ref.^32^ is fitted as:

20 $$\rho \left( T \right) = 4.31 \times 10^{{ - 8}} T^{2} + 9.804 \times 10^{{ - 3}} T + 7.62$$

**Fig. 15:**
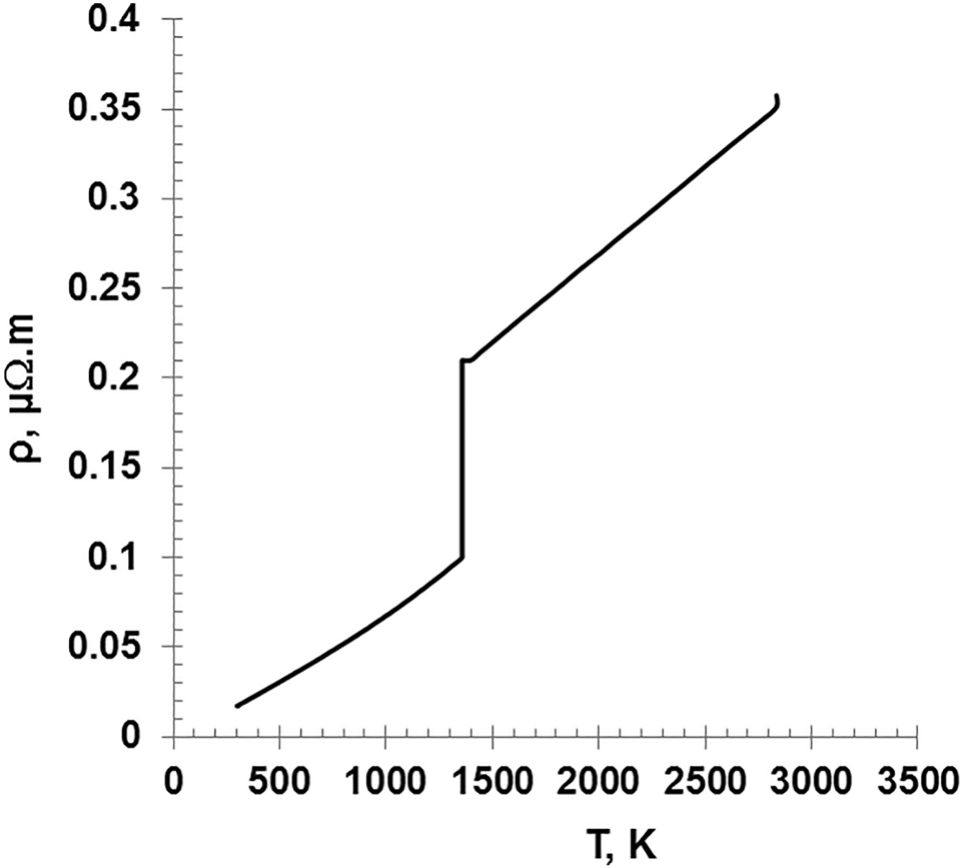
The variation of the wire resistivity as a function of the wire temperature.

**Fig. 16:**
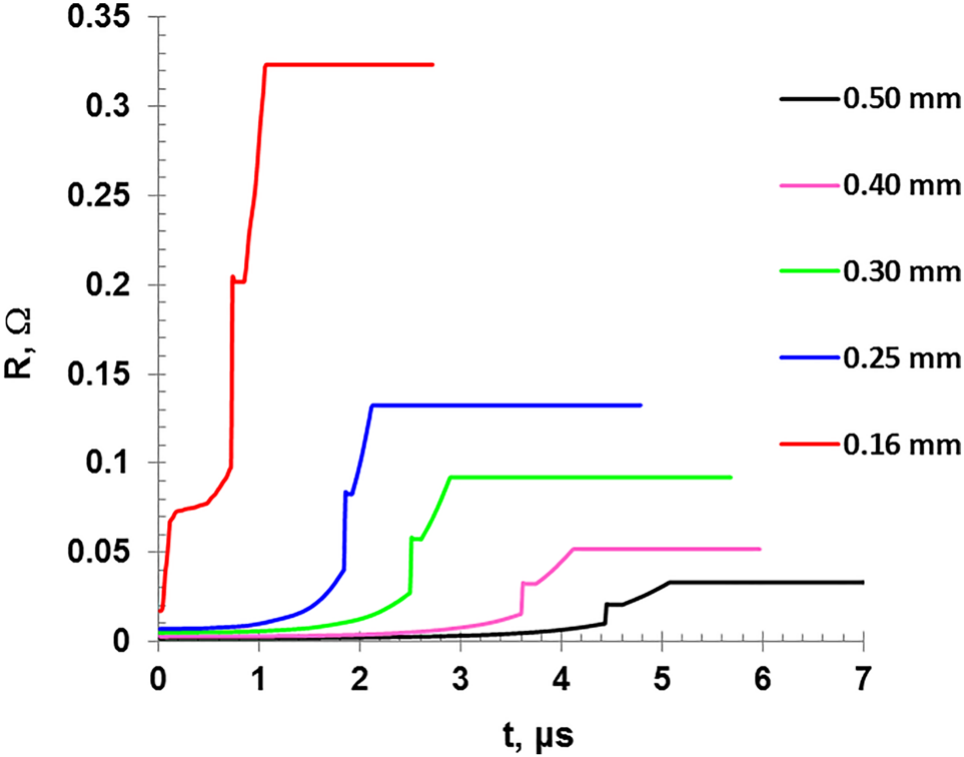
Variation of the wire resistance as a function of the discharge time.

Figure 15 shows the variation of the wire resistivity as a function of the wire temperature, as given by Eqs. 19 and 20. The figure illustrates two plateaus regions at the melting and boiling points. The behavior of the resistivity aligns with the previous researches studies^8,11^. On the other hand, Fig. 16 shows the wire resistance evolution as a function of the discharge time. The wire resistance is directly proportional to as the wire temperature which increases with time due to Ohmic heating and it is inversely proportional to the cross-sectional area. It is worth noting that by incorporating the wire expansion as illustrated in Fig. 12, the resistance of the wire will be reduced with about 9.2% compared to the result when the dimensional variation was taken into account.

## Conclusion

The pulsed electrothermal discharge experiment was used to study the copper wires’ explosion. The experiment used wires of different diameters in the range of 0.16–0.50 mm, and charging voltages in range between 2 and 6 kV. The discharge signal showed that the discharge current increases gradually at the beginning, and then a dip in the discharge current signals incorporated with a spike in the discharge voltage, which is higher for the thin wire, indicating that the wires were exploded, so that resistance is raised. Then the discharge current starts to increase again, indicating plasma formation.

The results showed that the time of explosion is inversely proportional to the charging voltage and it increases for the thicker wires due to the lower current density and the greater mass, which needs higher energy for the transition from the solid phase into the liquid phase and then to explosion, which finally transfers the wire into the vapor phase.

The effect of thermal expansion showed an increase in the wire length and the cross-sectional area by about 9.95% and 19.9%, respectively. Incorporation of the thermal expansion in the analysis affects the resistance calculations, reducing them by 9.2% as compared to the cases where the dimensional variation was not considered. Also, the variation of the specific heat capacity as a function of the wire temperature was obtained using Shomate equation. Although, the effect of energy deposition on wire diameter expansion behavior was studied previously, it is worth noting that the expansion behavior depends on the expansion time rate, which in turn depends on the rate of current rise.

Finally, it is worth noting that the specific action integral is almost the same of about 2.1 × 10^9^ A^2^ s/cm^4^ for all wire diameters, because it is considered as a material property, especially for current density below 10^8^ A/cm^2^.

Future research could explore several variables, such as the effect of the ambient gas pressure, composite materials, and using of multiple wires. The presented analysis which is related to thermal expansion and electrical resistance provides a base for other researches such as the wire array z-pinch and production of nanoparticles in pulsed power systems as examples.

## Data Availability

The data that support the findings of this study are available within the article.
